# Association of Hospital Payment Profiles With Variation in 30-Day Medicare Cost for Inpatients With Heart Failure or Pneumonia

**DOI:** 10.1001/jamanetworkopen.2019.15604

**Published:** 2019-11-15

**Authors:** Harlan M. Krumholz, Yongfei Wang, Kun Wang, Zhenqiu Lin, Susannah M. Bernheim, Xiao Xu, Nihar R. Desai, Sharon-Lise T. Normand

**Affiliations:** 1 Section of Cardiovascular Medicine, Department of Internal Medicine, Yale School of Medicine, New Haven, Connecticut; 2 Center for Outcomes Research and Evaluation, Yale-New Haven Hospital, New Haven, Connecticut; 3 Department of Health Policy and Management, Yale School of Public Health, New Haven, Connecticut; 4 Real World Analytic and Alliance, Janssen Scientific Affairs, Titusville, New Jersey; 5 Section of General Internal Medicine, Department of Internal Medicine, Yale School of Medicine, New Haven, Connecticut; 6 Department of Obstetrics, Gynecology and Reproductive Sciences, Yale School of Medicine, New Haven, Connecticut; 7 Department of Health Care Policy, Harvard Medical School, Boston, Massachusetts; 8 Department of Biostatistics, Harvard T.H. Chan School of Public Health, Boston, Massachusetts

## Abstract

**Importance:**

Some uncertainty exists about whether hospital variations in cost are largely associated with differences in case mix.

**Objective:**

To establish whether the same patients admitted with the same diagnosis (heart failure or pneumonia) at 2 different hospitals incur different costs associated with the hospital’s Medicare payment profile.

**Design, Setting, and Participants:**

This observational cohort study used Centers for Medicare & Medicaid Services (CMS) discharge data of patients with a principal diagnosis of heart failure (n = 1615) or pneumonia (n = 708) occurring between July 1, 2013, and June 30, 2016. Patients were individuals aged 65 years or older who were enrolled in Medicare fee-for-service Part A and Part B and were discharged from nonfederal, short-term, acute care or critical access hospitals in the United States. Data were analyzed from March 16, 2018, to September 25, 2019.

**Main Outcomes and Measures:**

The CMS heart failure and pneumonia payment measure cohorts were divided into 2 random samples. In the first sample, hospitals were classified into payment quartiles for heart failure and pneumonia. In the second sample, patients with 2 admissions for heart failure or pneumonia, one in a lowest-quartile hospital and one in a highest-quartile hospital more than 1 month apart, were identified. Standardized Medicare payments for these patients were compared for the lowest- and the highest-quartile payment hospitals.

**Results:**

The study sample included 1615 patients with heart failure (mean [SD] age, 78.7 [8.0] years; 819 [50.7%] male) and 708 with pneumonia (mean [SD] age, 78.3 [8.0] years; 401 [56.6%] male). The observed 30-day mortality rates for patients among lowest- compared with highest-payment hospitals were not significantly different. The median (interquartile range) hospital 30-day risk-standardized mortality rates were 8.1% (7.7%-8.5%) for heart failure and 11.3% (10.7%-12.1%) for pneumonia. The 30-day episode payment for hospitalization for the same patients at the lowest-payment hospitals was $2118 (95% CI, $1168-$3068; *P* < .001) lower for heart failure and $2907 (95% CI, $1760-$4054; *P* < .001) lower for pneumonia than at the highest-payment hospitals. More than half of the difference was associated with the payment during the index hospitalization ($1425 [95% CI, $695-$2154; *P* < .001] for heart failure and $1659 [95% CI, $731-$2588; *P* < .001] for pneumonia).

**Conclusions and Relevance:**

This study found that the same Medicare beneficiaries who were admitted with the same diagnosis to hospitals with the highest payment profiles incurred higher costs than when they were admitted to hospitals with the lowest payment profiles. The findings suggest that variations in payments to hospitals are, at least in part, associated with the hospitals independently of non–time-varying patient characteristics.

## Key Points

**Question:**

Is the amount that the Centers for Medicare & Medicaid Services pays hospitals associated with characteristics of the hospital independent of non–time-varying characteristics of patients?

**Findings:**

In this cohort study of 1615 Medicare beneficiaries hospitalized with heart failure and 708 beneficiaries hospitalized with pneumonia, the same patients admitted with the same diagnosis to hospitals in the highest Medicare payment quartile had higher costs than when they were admitted to hospitals in the lowest quartile.

**Meaning:**

Findings from this study suggest that hospitals operate at different cost levels independent of their patients.

## Teaser

This cohort study analyzes Centers for Medicare & Medicaid Services data for heart failure and pneumonia discharges to ascertain hospital payment profiles associated with hospitalization costs for Medicare beneficiaries.

## Introduction

A central focus in health care is the cost of care.^1^ The Centers for Medicare & Medicaid Services (CMS) introduced a publicly reported payment measure in 2016 for heart failure and pneumonia.^2,3^ This National Quality Forum–approved measure captured Medicare payments over a 30-day period starting at admission. The measure showed substantial variation in payments across hospitals, signaling that different hospitals have different payment profiles. For example, for heart failure—one of the most common causes of hospitalization for Medicare beneficiaries—the risk-standardized payments (RSPs) for the 30-day episode of care, including hospitalization and postacute care, among hospitals ranged from $11 652 to $21 819 in the July 1, 2013, to June 30, 2016, public reporting period.^4^ The amount that CMS pays, not including policy adjustments for geography and medical education, is a marker of resource use, including the intensity and length of treatment during the initial hospitalization and the events and services in the postacute period.^3^

The premise of characterizing the variation in CMS payments is that it reflects the various proclivities of the hospital and its postacute ecosystem.^5^ A concern, however, is that these measures instead may reflect differences in case mix.^6,7^ Although the payment measures are adjusted for demographic and clinical factors, unmeasured variations in patient populations of different hospitals may be associated with resource use in and out of the hospital. However, disentangling the intrinsic patterns of resource use from patient factors is difficult. The implications are profound because if differences in cost arise from differences in case mix, then lower-cost institutions should not be commended, emulated, or rewarded.^8^ Alternatively, if variations are associated with different hospital and postacute patterns of care, then the lower payment profile, its association with quality, and its association with patient outcomes should be understood.

The objective of this cohort study was to establish whether the same patients with the same diagnosis (heart failure or pneumonia) admitted to 2 different hospitals incurred different costs associated with the hospital’s Medicare payment profile. We chose heart failure and pneumonia because these measures are publicly reported, and patients with these conditions are often admitted more than once with the same diagnosis. To isolate the hospital factors from the patient factors, including unmeasured largely non–time-varying factors such as social context and economic class, we leveraged the observation that many patients have multiple admissions with these diagnoses at different hospitals. Accordingly, we characterized hospitals into quartiles based on 30-day payment performance within the 3-year measurement period. Next, using a separate sample of patients admitted with heart failure or pneumonia, we identified those admitted to hospitals located in the lowest and highest quartiles of 30-day payment performance. The prespecified focus of this study was on hospitals in the lowest and highest payment quartiles to ensure a marked contrast in payments between the comparison hospitals. We identified the difference in 30-day payments to these hospitals and the variation in payments. As mentioned, the principal aim was to ascertain whether the same patients with the same diagnosis at hospitals classified differently on the payment measure would have different 30-day CMS payments.

## Methods

### Data and Cohort

In this cohort study, we analyzed the data sets used in the 2017 CMS public reporting for the 30-day episode of care payment measure for heart failure and pneumonia.^9^ We obtained approval for this study from the Yale University Human Investigation Committee along with a waiver of the requirement for participant informed consent; the waiver was granted because it was not feasible to obtain consent and the data were reused in accordance with our data use agreement with CMS. This study followed the Strengthening the Reporting of Observational Studies in Epidemiology (STROBE) reporting guideline.

The analysis included patients with a principal discharge diagnosis of heart failure or pneumonia occurring between July 1, 2013, and June 30, 2016, from nonfederal, short-term, acute care or critical access hospitals in the United States. These discharges involved patients aged 65 years or older who were enrolled in Medicare fee-for-service Part A and Part B for the 12 months before the date of admission and during the index admission and who were not transferred from another acute care facility. Reasons for exclusion were being discharged alive on the day of admission or the following day for patients who were not transferred to another acute care facility, being discharged against medical advice, being transferred to a federal hospital, lacking index diagnosis-related group (DRG) weight in which the facility or practitioner received no payment, having incomplete follow-up on administrative data in the 30-day episode of care, or being enrolled in the Medicare hospice program any time in the 12 months before or at admission. For the heart failure cohort, we excluded as part of the heart failure definition admissions in which the patient had a procedure code for a left ventricular assist device implant or a heart transplant either during the index admission or in the 12 months before the index admission.

For each condition (heart failure and pneumonia), we randomly split the discharges into 2 groups, which were stratified by hospital. In the first half of the discharges, we randomly selected 1 discharge if a patient had more than 1 discharge in a given year, which is consistent with the payment measure cohort derivation. Next, we calculated the RSP in a manner consistent with the CMS heart failure and pneumonia payment measures, which remove geographic and policy adjustments. Hospitals were classified into quartiles of payment performance. This sample constituted the performance classification cohort used to define hospital payment performance. In this cohort, the median payments in the upper and lower quartiles were $13 789 and $16 651 for heart failure and $13 606 and $18 382 for pneumonia.

We used the other half of the discharges in each condition to construct the sample of patients who were hospitalized twice for the same condition during the study period, once at institutions in the lowest payment performance quartile and once at institutions in the highest payment performance quartile (eFigure 1 in the Supplement). For patients who had more than 1 admission in the same quartile, only 1 admission was randomly selected. This second sample constituted the study cohort used to evaluate payments for the same patients with the same diagnosis admitted to hospitals with different payment performance.

### Outcome and Hospital Payment Performance

The primary outcome was the Medicare total payments in the 30 days after index admission. Payment was determined according to the methods of the CMS publicly reported payment measures.^9^ The outcome captured all payments within a 30-day period from the date of the index admission across multiple care settings and services, including inpatient, outpatient, skilled nursing, home health, hospice, physician or clinical, laboratory or ambulance, durable medical equipment, prosthetics or orthotics, and supplies. To help standardize the implications of factors unrelated to clinical care, we removed geographic differences and policy adjustments in payment rates from the total payment for individual services.^10^ When geographic differences in payments could not be removed, we calculated the means across geographic areas.^11^ We also winsorized the total payment at the higher end for payments more than the 99 percentile to the 99 percentile value.

To calculate the hospital-specific RSP, as with the publicly reported measure, we modeled payment, using the performance classification sample, as a function of patient age and clinically relevant comorbidities with an intercept for the hospital-specific random effect. We then calculated the RSPs as the ratio of the estimated payment to the expected payment at a given hospital, multiplied by the national mean payment. The estimated payment for each hospital was calculated using its case mix and an estimated hospital-specific factor for that hospital. The expected payment for each hospital was estimated using its case mix and the mean hospital factor. Details of these calculations can be found in the technical reports for CMS publicly reported heart failure and pneumonia payment measures.^9^ Next, we grouped hospitals into quartiles according to their RSPs in the performance classification sample; quartile 1 included the low-payment hospitals (lowest RSPs), and quartile 4 included the high-payment hospitals (highest RSPs).

### Statistical Analysis

We reported the patient and hospital characteristics for the final study sample in each condition. We compared the patient characteristics between the study sample and the exclusions. For the hospital characteristics, we used the 2015 American Hospital Association annual survey database.^12^

To assess the similarity between paired admissions, we compared the admission characteristics between quartile arms. We reported the percentages of first admissions that occurred at the lowest-quartile hospitals. We also compared the percentage of admissions with percutaneous coronary intervention, coronary artery bypass graft, or placement of an implantable cardioverter defibrillator for patients with heart failure (assessing procedures that might be associated with cost); the percentage of unplanned readmissions within 30 days of the discharge; and the length of stay for both heart failure and pneumonia. In addition, we reported risk-standardized 30-day mortality rates using previously described methods.^13,14^

For each condition, we tested whether payment for patients who had an admission to a hospital in the low-payment group was different from payment for patients admitted to a hospital in the high-payment group. To assess the significance of difference in payment, we calculated the difference in the total payment and subcategories of payment during the episode of care between the admissions on each pair. We used paired, 2-tailed *t* test or Wilcoxon signed rank test to calculate the *P* values. For the subcategories of payment, we considered payment in the index admission; the facility and physician payment during the readmission; and the postacute care setting, including emergency department, home health agency, hospice, nonacute inpatient, outpatient physician visits, readmission facility, readmission physician, skilled nursing facility, and miscellaneous categories. We adjusted for multiplicity of testing using the Bonferroni procedure. With 13 primary 2-sided tests with an overall error of 0.05, we required *P* < .004 to declare the differences statistically significant. The statistical analyses were performed with SAS, version 9.4 (SAS Institute Inc) and STATA/MP, version 13.1 (StataCorp LLC) software. Data were analyzed from March 16, 2018, to September 25, 2019.

## Results

### Hospital Performance Classification

The study sample development is shown in eFigure 1 in the Supplement. In the study period, 1 087 261 discharges met the inclusion or exclusion criteria for heart failure, and 1 372 323 discharges met the criteria for pneumonia. Of these discharges, 543 631 (50.0%) for heart failure and 686 162 (50.0%) for pneumonia were randomly selected for the payment classification sample, which was analyzed for the classification of hospital payment profiles. After the inclusion or exclusion of random selection of 1 admission within a year was applied, as required in the payment measure, the payment classification samples had 498 153 discharges for heart failure and 657 155 discharges for pneumonia for the calculation of RSPs. The distribution of hospital RSPs for heart failure and pneumonia in the payment classification sample is shown in eFigure 2 in the Supplement. The median (interquartile range [IQR]) hospital RSPs were $15 783 ($15 241-$16 427) for heart failure and $16 774 ($15 751-$17 764) for pneumonia. The median (IQR) hospital 30-day risk-standardized mortality rates were 8.1% (7.7%-8.5%) for heart failure and 11.3% (10.7%-12.1%) for pneumonia.

### Study Sample

The other half of the random sample, which produced the study sample, included 543 630 discharges for heart failure and 686 161 discharges for pneumonia.

The study sample comprised 1615 patients with heart failure (mean [SD] age, 78.7 [8.0] years; 819 [50.7%] male) and 708 with pneumonia (mean [SD] age, 78.3 [8.0] years; 401 [56.6%] male) who were admitted to low-payment and high-payment hospitals. The characteristics of these patients are shown in eTable 1 in the Supplement, with no differences observed between patients in low- and high-payment hospitals except for history of infection (43 [2.7%] vs 29 [1.8%]) in the heart failure cohort and for protein-calorie malnutrition (138 [19.5%] vs 159 [22.5%]), iron deficiency and other or unspecified anemias and blood disease (458 [64.7%] vs 501 [70.8%]), dementia and senility (247 [34.9%] vs 281 [39.7%]), heart infection or inflammation except rheumatic (21 [3.0%] vs 31 [4.4%]), other eye disorders (167 [23.6%] vs 142 [20.1%]), and traumatic amputations and complications and other injuries (324 [45.8%] vs 362 [51.1%]) in the pneumonia cohort.

The patient demographic characteristics between the study sample and those excluded from the sample in the heart failure and pneumonia cohorts are shown in eTable 2 in the Supplement. Table 1 shows some selected patient characteristics during the paired admissions between the first quartile and last quartile for both heart failure and pneumonia. For heart failure, the admissions in low-payment hospitals compared with admissions in high-payment hospitals were significantly less likely to have been provided with percutaneous coronary intervention (8 [0.5%] vs 24 [1.5%]; *P* = .005) or an implantable cardioverter defibrillator (12 [0.7%] vs 47 [2.9%]; *P* < .001). The median (IQR) length of stay (heart failure: 4 [3-6] days vs 5 [3-7] days; pneumonia: 4 [3-6] days vs 5 [3-7] days) and readmission rate (heart failure: 440 [27.2%] vs 450 [27.9%]; standardized mean difference [SMD], −0.0139 [95% CI, −0.0806 to 0.0529]; pneumonia: 160 [22.6%] vs 161 [22.7%]; SMD, −0.0034 [95% CI, −0.1043 to 0.0976]) were not significantly different between the low-payment and high-payment hospitals for heart failure and pneumonia.

**Table 1.** Selected Patient Characteristics and Outcomes Among Paired Admissions

Variable	No. (%)			Standardized Mean Difference (95% CI)
	Total	Admissions in Low-Payment Hospitals	Admissions in High-Payment Hospitals	
Heart failure				
No. of admissions	3230	1615	1615	NA
Age, mean (SD), y	79 (8.0)	79 (8.0)	79 (8.1)	−0.0052 (−0.0115 to 0.0010)
With PCI during hospitalization	32 (0.9)	8 (0.5)	24 (1.5)	−0.1001 (−0.1695 to −0.0308)
CABG during hospitalization	5 (0.2)	0	5 (0.3)	−0.0788 (−0.1478 to −0.0098)
ICD during hospitalization	59 (1.8)	12 (0.7)	47 (2.9)	−0.1623 (−0.2320 to −0.0926)
LOS, d				
Mean (SD)	6 (4.5)	5 (4.5)	6 (4.6)	−0.0926 (−0.1561 to −0.0290)
Median (IQR)	4 (3-7)	4 (3-6)	4 (3-7)	NA
Readmission within 30 d of discharge	890 (27.6)	440 (27.2)	450 (27.9)	−0.0139 (−0.0806 to 0.0529)
Distance between patient home and hospital, miles				
Mean (SD)	113 (336.5)	97 (300.9)	129 (367.9)	−0.0958 (−0.1595 to −0.0322)
Median (IQR)	13 (4-41)	11 (3-9)	16 (6-34)	NA
30-d Mortality from admission	176 (5.5)	88 (5.5)	88 (5.5)	0.0000 (−0.0709 to 0.0709)
Pneumonia				
No. of admissions	1416	708	708	NA
Age, mean (SD), y	78 (8.0)	78 (8.0)	78 (8.0)	−0.0067 (−0.0167 to 0.0033)
Primary sepsis and secondary pneumonia	285 (20.1)	109 (15.4)	176 (24.9)	−0.2375 (−0.3401 to −0.1349)
LOS, d				
Mean (SD)	6 (4.2)	5 (3.9)	6 (4.5)	−0.1950 (−0.2944 to −0.0957)
Median (IQR)	5 (3-7)	4 (3-6)	5 (3-7)	NA
Readmission	321 (22.7)	160 (22.6)	161 (22.7)	−0.0034 (−0.1043 to 0.0976)
Distance between patient home and hospital, miles				
Mean (SD)	102 (291.3)	82 (264.8)	122 (314.4)	−0.1392 (−0.2433 to −0.0350)
Median (IQR)	17 (4-43)	11 (0.3-28)	26 (8-54)	NA
30-d Mortality from admission	84 (5.9)	39 (5.5)	45 (6.4)	−0.0359 (−0.1433 to 0.0716)

Abbreviations: CABG, coronary artery bypass graft; ICD, implantable cardioverter defibrillator; IQR, interquartile range; LOS, length of stay; NA, not applicable; PCI, percutaneous coronary intervention.

The median (IQR) time between paired admissions was 204 (94-396) days for heart failure and 225 (104-414) days for pneumonia; the percentages of first admissions that occurred at the lowest-quartile hospitals were 52% for heart failure and 52% for pneumonia. No significant difference was observed in mean [SD] days between the paired admissions among patients first admitted to the low-payment hospitals and among patients first admitted to the high-payment hospitals (heart failure: 276.4 [220.5] vs 267.6 [221.8]; *P* = .26; pneumonia: 297.8 [236.3] vs 284.9 [224.2]; *P* = .29).

The observed 30-day mortality rates for patients in low-payment hospitals compared with patients in high-payment hospitals were not significantly different for heart failure (5.4% vs 5.4%; *P* = >.99; odds ratio [OR], 1.000; 95% CI, 0.736-1.359) or pneumonia (5.5% vs 6.4%; *P* = .51; OR, 1.154; 95% CI, 0.735-1.819).

### Hospital and Payment Comparisons

Compared with the high-payment hospitals, the low-payment hospitals tended to be non-teaching (heart failure: 270 [49.2%] vs 377 [67.4%]; *P* < .001; pneumonia: 173 [52.1%] vs 307 [80.6%]; *P* < .001), smaller (<200 beds, heart failure: 212 [38.6%] vs 375 [67.1%]; *P* < .001; pneumonia: 154 [46.4%] vs 315 [82.7%]; *P* < .001), safety net (heart failure: 98 [17.9%] vs 159 [28.4%]; *P* < .001; pneumonia: 73 [21.9%] vs 126 [33.1%]; *P* < .001), rural (heart failure: 34 [6.2%] vs 87 [15.6%]; *P* < .001; pneumonia: 38 [11.5%] vs 111 [29.1%]; *P* < .001), or government facilities (heart failure: 63 [11.5%] vs 123 [22.0%]; *P* < .001; pneumonia: 44 [13.3%] vs 100 [26.3%]; *P* = .001) for both conditions (Table 2). The low-quartile, compared with the high-quartile, hospitals were also less likely to be trauma centers (heart failure: 287 [51.3%] vs 293 [53.4%]; *P* = .49; pneumonia: 175 [45.9%] vs 181 [54.5%]; *P* = .02) and to have cardiac surgical procedure capability (heart failure: 238 [42.6%] vs 373 [67.9%]; *P* < .001; pneumonia: 92 [24.2%] vs 198 [59.6%]; *P* < .001).

**Table 2.** Hospital Characteristics in the Heart Failure and Pneumonia Study Samples, Overall and in Low-Payment and High-Payment Hospitals

Variable	No. (%)			*P* Value
	Total	Low-Payment Hospitals	High-Payment Hospitals	
Heart Failure				
All	1121	566	555	NA
Teaching hospital				<.001
No	647 (58.4)	377 (67.4)	270 (49.2)	
Yes	461 (41.6)	182 (32.6)	279 (50.8)	
Safety-net hospital^a^				<.001
No	851 (76.8)	400 (71.6)	451 (82.2)	
Yes	257 (23.2)	159 (28.4)	98 (17.9)	
Census region				.01
Northeast	176 (15.9)	71 (12.7)	105 (19.1)	
Midwest	285 (25.7)	148 (26.5)	137 (24.9)	
South	432 (38.9)	226 (40.4)	206 (37.5)	
West	207 (18.7)	107 (19.1)	100 (18.2)	
Other	8 (0.7)	7 (1.3)	1 (0.2)	
Core-based statistical area				<.001
Rural	121 (10.9)	87 (15.6)	34 (6.2)	
Urban	987 (89.1)	472 (84.4)	515 (93.8)	
No. of beds				<.001
<200	587 (52.9)	375 (67.1)	212 (38.6)	
200 to <300	195 (17.6)	84 (15.0)	111 (20.2)	
300 to <400	127 (11.5)	33 (5.9)	94 (17.1)	
400 to <500	64 (5.8)	25 (4.5)	39 (7.1)	
≥500	135 (12.2)	42 (7.5)	93 (16.9)	
Ownership				<.001
Government	186 (16.8)	123 (22.0)	63 (11.5)	
Not-for-profit	738 (66.6)	360 (64.4)	378 (68.9)	
For-profit	184 (16.6)	76 (13.6)	108 (19.7)	
Cardiac facility				<.001
CABG	611 (55.1)	238 (42.6)	373 (67.9)	
Catheter laboratory only	138 (12.5)	80 (14.3)	58 (10.6)	
Other	359 (32.4)	241 (43.1)	118 (21.5)	
Trauma center				.49
No	528 (47.7)	272 (48.7)	256 (46.6)	
Yes	580 (52.4)	287 (51.3)	293 (53.4)	
Any PCI done in the study period				<.001
No	587 (52.4)	383 (67.7)	204 (36.8)	
Yes	534 (47.6)	183 (32.3)	351 (63.2)	
Any CABG done in the study period				<.001
No	894 (79.8)	505 (89.2)	389 (70.1)	
Yes	227 (20.3)	61 (10.8)	166 (29.9)	
Any ICD done in the study period				<.001
No	551 (49.2)	372 (65.7)	179 (32.3)	
Yes	570 (50.9)	194 (34.3)	376 (67.8)	
Pneumonia				NA
All	726 (100.0)	387 (100.0)	339 (100.0)	
Teaching hospital				<.001
No	480 (67.3)	307 (80.6)	173 (52.1)	
Yes	233 (32.7)	74 (19.4)	159 (47.9)	
Safety-net hospital^a^				.001
No	514 (72.1)	255 (66.9)	259 (78.0)	
Yes	199 (27.9)	126 (33.1)	73 (21.9)	
Census region				.49
Northeast	95 (13.3)	44 (11.6)	51 (15.4)	
Midwest	175 (24.5)	92 (24.2)	83 (25.0)	
South	339 (47.6)	187 (49.1)	152 (45.8)	
West	103 (14.5)	57 (14.9)	46 (13.9)	
Other	1 (0.1)	1 (0.3)	0 (0)	
Core-based statistical area				<.001
Rural	149 (20.9)	111 (29.1)	38 (11.5)	
Urban	564 (79.1)	270 (70.9)	294 (88.6)	
No. of beds				<.001
<200	469 (65.8)	315 (82.7)	154 (46.4)	
200 to <300	89 (12.5)	26 (6.8)	63 (18.9)	
300 to <400	57 (7.9)	15 (3.9)	42 (12.7)	
400 to <500	40 (5.6)	10 (2.6)	30 (9.0)	
≥500	58 (8.1)	15 (3.9)	43 (12.9)	
Ownership				.001
Government	144 (20.2)	100 (26.3)	44 (13.3)	
Not-for-profit	427 (59.9)	216 (56.7)	211 (63.6)	
For-profit	142 (19.9)	65 (17.1)	77 (23.2)	
Cardiac facility				<.001
CABG	290 (40.7)	92 (24.2)	198 (59.6)	
Catheter laboratory only	89 (12.5)	52 (13.7)	37 (11.1)	
Other	334 (46.8)	237 (62.2)	97 (29.2)	
Trauma center				.02
No	357 (50.1)	206 (54.1)	151 (45.5)	
Yes	356 (49.9)	175 (45.9)	181 (54.5)	
Any PCI done in the study period				<.001
No	596 (82.1)	360 (93.0)	236 (69.6)	
Yes	130 (17.9)	27 (6.9)	103 (30.4)	
Any CABG done in the study period				.16
No	714 (98.4)	383 (98.9)	331 (97.6)	
Yes	12 (1.7)	4 (1.0)	8 (2.4)	
Any ICD done in the study period				<.001
No	670 (92.3)	374 (96.6)	296 (87.3)	
Yes	56 (7.7)	13 (3.4)	43 (12.7)	

Abbreviations: CABG, coronary artery bypass graft; ICD, implantable cardioverter defibrillator; NA, not applicable; PCI, percutaneous coronary intervention.

^a^ Percentages may not total 100% because of rounding. Thirteen hospitals in the heart failure study sample and 6 hospitals in the pneumonia study sample could not be matched with American Hospital Association 2015 survey data.

**Figure 1.**
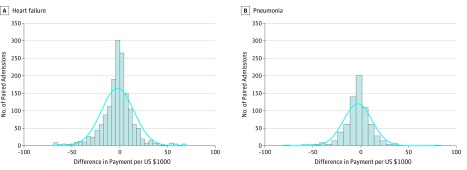
Distribution of the Differences in Payment Between Paired Admissions for Heart Failure and Pneumonia The solid curve is the normal density of the distribution. Difference is the payment in lowest quartile minus the payment in highest quartile.

**Figure 2.**
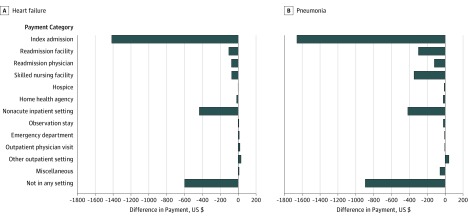
Sources of Difference in Payments Between Low-Payment and High-Payment Hospitals for Heart Failure and Pneumonia

The same patients hospitalized for the same diagnoses at low- and high-payment hospitals had significantly different total payment (Table 3). The distributions of the differences are shown in Figure 1. For heart failure, for the same patients, hospitalization at a low-payment hospital was associated with a $2118 (95% CI 1168-3068; *P* < .001) lower total episode payment over 30 days, compared with hospitalization at a high-payment hospital. For pneumonia, for the same patients, hospitalization at the low-payment hospital was associated with a $2907 lower total episode payment (95% CI, $1760-$4054; *P* < .001). More than half of the difference was associated with the payment during the index admission for both conditions ($1425 [95% CI, $695-$2154; *P* < .001] for heart failure and $1659 [95% CI, $731-$2588; *P* < .001] for pneumonia). The comparison for the subcategories of payments is shown in Table 3 and Figure 2. The results were not different regardless of the sequence of the hospitalization (whether admitted to the low- or high-payment hospital first) or whether we adjusted for the sequence. Furthermore, adjusting for differences in the sepsis diagnosis for pneumonia did not substantively change the results. In eTable 3 in the Supplement, we present the specific DRG codes for the index hospitalization for low- and high-payment hospitals.

**Table 3.** Payments Between the Paired Admissions in the Low-Payment and High-Payment Hospitals

Variable	No. of Pairs	Mean of Payment		Difference in Payment Between Low-Payment and High-Payment Hospitals, Mean (95% CI)	*P* Value
		Admissions in Low-Payment Hospitals	Admissions in High-Payment Hospitals		
Heart Failure					
Total	1615	14 966	17 085	−2118 (−3068 to −1168)	<.001
Total, winsorized	1615	14 690	16 842	−2153 (−2983 to −1323)	<.001
Index admission	1615	10 596	12 020	−1425 (−2154 to −695)	<.001
Readmission, facility	1615	1454	1558	−105 (−504 to 295)	.50
Readmission, physician	1615	239	314	−75 (−135 to −14)	.02
Skilled nursing facility	1615	1346	1419	−73 (−323 to 177)	.57
Hospice	1615	116	117	−1 (−46 to 44)	.96
Home health agency	1615	171	188	−17 (−52 to 18)	.35
Nonacute inpatient settings	1615	205	640	−435 (−650 to −221)	<.001
Observational stay	1615	96	88	8 (−67 to 84)	.34
Emergency department	1615	96	108	−12 (−34 to 9)	.26
Outpatient physician visits	1615	118	138	−20 (−50 to 10)	.96
Other outpatient settings	1615	282	250	32 (−42 to 106)	.72
Miscellaneous	1615	246	235	10 (−51 to 72)	.52
Not in any setting	1615	2672	3271	−599 (−976 to −222)	.001
Pneumonia					
Total	708	14 603	17 510	−2907 (−4054 to −1760)	<.001
Total, winsorized	708	14 599	17 398	−2799 (−3898 to −1700)	<.001
Index admission	708	10 155	11 815	−1659 (−2588 to −731)	<.001
Readmission facility	708	854	1150	−296 (−649 to 58)	.10
Readmission physician	708	134	259	−125 (−189 to −61)	.001
Skilled nursing facility	708	1987	2336	−348 (−843 to 146)	.17
Hospice	708	100	111	−11 (−75 to 52)	.73
Home health agency	708	149	171	−22 (−74 to 30)	.40
Nonacute inpatient settings	708	423	851	−428 (−817 to −39)	.03
Observational stay	708	60	40	21 (−36 to 77)	.35
Emergency department	708	99	106	−7 (−42 to 29)	.71
Outpatient physician visits	708	142	147	−5 (−51 to 41)	.82
Other outpatient settings	708	293	251	42 (−71 to 155)	.62
Miscellaneous	708	205	263	−58 (−156 to 40)	.89
Not in any setting	708	3389	4283	−894 (−1557 to −231)	.008

## Discussion

In this cohort study, the same patients with the same diagnosis admitted to hospitals with different Medicare payment profiles incurred different costs. For example, a patient admitted for heart failure within 3 years to a low-payment hospital and a high-payment hospital incurred a significantly higher CMS payment for hospitalization and postacute care at the high-payment hospital regardless of the sequence of the hospitalizations. By studying patients who were admitted twice for the same diagnosis at hospitals with different payment profiles, we were able to better isolate the association of the hospital and its ecosystem from other factors, such as social context.

We believe this study extends the literature in important ways. We were able to isolate a hospital factor in the variation in payments to hospitals. This finding suggests that the variation in RSP is, in part, associated with factors that are unrelated to the patients. In particular, this study is able to hold constant the social determinants of health that were not expected to change between the 2 admissions. We compared the same people at 2 different hospitals so that behaviors, social context, and demographic characteristics, including race/ethnicity, were the same. The assumption of this design was that these other factors, which have been implicated in patient risk but can be hard to measure directly, were now similar in the comparison groups. We found marked differences in payment depending on which hospital the patient received care at. No evidence was found that lower-payment hospitals had higher mortality rates.

In addition, this study appears to support the CMS measure of classifying hospitals on the basis of their payments, holding constant their case mix. The hospitals classified as high payment by the CMS measure indeed had higher payment when unmeasured, non–time-varying characteristics of patients, such as many social determinants, were held constant in this study design. Furthermore, the difference between low-payment and high-payment hospitals was only slightly different from the variation seen in the by-quartile comparison of all patients.

Previous studies have assessed the variation in spending across hospitals. For example, a marked and significant variation in the use of the intensive care unit was found among patients with heart failure.^15^ In a diverse group of 341 hospitals in the United States, the use of an intensive care unit for heart failure varied from 0% to 88%.^15^ Significant hospital variations in readmissions and in the use of skilled nursing facilities for heart failure and pneumonia have been researched as well.^16,17^ Other studies have long reported variations in hospital-level resource consumption.^18^ With such variations, it is not surprising that different hospital resource-consumption profiles associated with hospitalizations exist. We believe the present investigation adds to the published work by demonstrating that the payment profile seemed unrelated to the patient, as the patient was held constant in this study.

The measure of total payments in this study included payments for the index hospitalization and all subsequent payments up to 30 days from admission. These payments are not costs, and use of DRG-based payment can limit the sensitivity of measuring resource use; however, patients with heart failure or pneumonia can end up having different DRGs (despite having the same principal discharge diagnosis) depending on their clinical scenario (eg, whether complications or major complications arise as a result of care received during the index admission). For instance, in the pneumonia sample, the 6 most commonly reported DRGs were 177 (respiratory tract infections and inflammations with major complication or comorbidity [MCC]), 178 (respiratory tract infections and inflammations with complication or comorbidity [CC]), 193 (simple pneumonia and pleurisy with MCC), 194 (simple pneumonia and pleurisy with CC), 195 (simple pneumonia and pleurisy without CC or MCC), and 871 (septicemia or severe sepsis without mechanical ventilation, more than 96 hours without MCC). The DRGs had a relative payment weight that ranged from 0.71 to 1.90 in the 2016 Medicare fee schedule, and DRG 177 accounted for 11.2% of the sample, 178 for 7.7%, 193 for 17.4%, 194 for 27.8%, 195 for 10.5%, and 871 for 19.0%. Hence, these DRGs could result in a 2.7-fold (ie, 1.90/0.71) difference in payments for hospital inpatient facility fees alone. Therefore, use of the DRG-based payments can still reasonably capture some variation in resource use. Moreover, DRG-based payment only affects hospital inpatient facility fees, which are only 1 component of the 30-day total payment that we report. Other resource use, such as physician professional fees during the index hospital stay and after discharge, subsequent skilled nursing facility care, subsequent home health visit, and subsequent admissions, are also associated with the substantial proportions of the 30-day total payment and the variations in payment.

The higher-cost institutions for heart failure have more procedures. We assessed 30-day payments, and any hospitals without the facilities in which to perform procedures could have transferred or referred patients for procedures, and those payments would have been included. Thus, the facilities at the initial hospital should not have restricted access to procedures within 30 days of admission.

This study may have implications for efforts to understand the cost of care. The comparison of payments has been challenging in observational studies because of concerns that differences in case mix are associated with differences in resource use. This study suggests that a hospital factor extended through the episode of care with respect to payment. In addition, no dominant source of payment difference was found that characterized the high-payment hospitals, and the general patterns were not consistent across conditions. We did not evaluate whether cost was associated with marginal benefit; the focus of this study was on identifying a distinct profile of hospitals on the basis of Medicare payments for the same condition. Moreover, we found that lower cost was not associated with a higher rate of deaths and that higher cost was not associated with better outcomes.

The lower-cost hospitals may represent achievable benchmarks and identify opportunities for reducing cost. For heart failure and pneumonia, the difference in the use of procedures was small. Lower readmission rates at low-payment hospitals were present for heart failure, which may have reduced payments in the institutions with low use. Significantly lower payments for hospitalization and postacute care were made for heart failure, and significantly lower costs for hospitalization were incurred for pneumonia. Research is needed to identify and address cultural and financial factors in resource use that might affect these different payment profiles. Nevertheless, the idea that lower costs are achievable may provide the impetus to investigate new strategies rather than simply resist the possibility that efficiencies can be achieved.

### Limitations

The study has several limitations. First, it was focused on a subgroup of all patients with these diagnoses. The patients were not typical in that, by design, they had hospitalizations for the same diagnoses at institutions with different payment profiles. However, this cohort enabled us to make the comparison among hospitals without concern about differences in patient case mix. Although patients in the study sample may not be representative of all patients, we believe the hospital behavior was accurately captured. The key point to consider is whether the behavior of the hospitals toward patients in the sample was generalizable to all of their patients, and we had no reason to believe that the hospitals treated these patients differently from the way they treated other patients. Also interesting and reassuring about generalizability was that the difference in payments in this select sample was as expected from the CMS measure based on all of the patients. Second, the focus of the study was on payment, and the CMS payment was not directly a proxy for hospital and postacute cost and resource use. It represented the actual cost to CMS and its related intensity and length of care.

Third, the sequence of the admissions was slightly unequal, with 52% of the first admissions being to low-payment hospitals. However, the difference was small, and adjusting for the sequence did not substantively affect the results. Fourth, this study was observational, and excluding the possibility of confounding was not possible. In this case, the confounding might occur not by differences in the patients but in the severity of their condition at the different hospitals. We lacked clinical details on the admissions, and some unmeasured differences were likely in the clinical circumstances of patients admitted to hospitals in the different tiers of payment performance. The similarities of the measurable characteristics of the patients and the lack of major differences in outcomes, particularly for heart failure, suggest no marked differences in clinical severity. The slight difference in mortality for pneumonia could be associated with unmeasured differences in admission or processes during the hospitalization. The finding for heart failure was close to that for pneumonia, and the mortality rates in heart failure were identical between the 2 types of hospitals.

## Conclusions

This cohort study found that when the same patients with the same diagnosis were admitted to hospitals with the highest or lowest level of resource use, as indicated by payment levels, the hospitals with the highest payment profiles incurred significantly higher Medicare costs over a 30-day episode of care. These findings suggest that variation in payments to hospitals are, at least in part, associated with the hospitals and their ecosystems independently of non–time-varying patient characteristics.

## Notes

Supplement. **eFigure 1.** Construction of the Study Cohort: Heart Failure (Part A) and Pneumonia (Part B) **eFigure 2.** The Distribution of Hospital Risk-Standardized Payments in the Performance-Classification Sample **eTable 1.** Patient Admission Characteristics in the Heart Failure and Pneumonia Study Samples, Overall and in Low-Payment and High-Payment Hospitals **eTable 2.** Patient Demographics Between the Study Sample and the Exclusions in the Heart Failure and Pneumonia Cohorts **eTable 3. **Diagnosis-Related Group Codes for the Index Hospitalization for Low-Payment and High-Payment Hospitals
