# Supplementary material for: Association of Hospital Payment Profiles With Variation in 30-Day Medicare Cost for Inpatients With Heart Failure or Pneumonia
Source: JAMA Netw Open. 2019 Nov 15;2(11):e1915604. doi: 10.1001/jamanetworkopen.2019.15604 (PMC6902811; doi:10.1001/jamanetworkopen.2019.15604)
Supplement: Supplement. — eFigure 1. Construction of the Study Cohort: Heart Failure (Part A) and Pneumonia (Part B) eFigure 2. The Distribution of Hospital Risk-Standardized Payments in the Performance-Classification Sample eTable 1. Patient Admission Characteristics in the Heart Failure and Pneumonia Study Samples, Overall and in Low-Payment and High-Payment Hospitals eTable 2. Patient Demographics Between the Study Sample and the Exclusions in the Heart Failure and Pneumonia Cohorts eTable 3. Diagnosis-Related Group Codes for the Index Hospitalization for Low-Payment and High-Payment Hospitals [file jamanetwopen-2-e1915604-s001.pdf]

Supplementary Online Content

Krumholz HM, Wang Y, Wang K, et al. Association of hospital payment profiles with variation in 30-day Medicare cost for inpatients with heart failure or pneumonia. *JAMA Netw Open.* 2019;2(11):e1915604. doi:10.1001/jamanetworkopen.2019.15604

eFigure 1. Construction of the Study Cohort: Heart Failure (Part A) and Pneumonia (Part B)

eFigure 2. The Distribution of Hospital Risk-Standardized Payments in the Performance-Classification Sample

eTable 1. Patient Admission Characteristics in the Heart Failure and Pneumonia Study Samples, Overall and in Low-Payment and High-Payment Hospitals

eTable 2. Patient Demographics Between the Study Sample and the Exclusions in the Heart Failure and Pneumonia Cohorts

eTable 3. Diagnosis-Related Group Codes for the Index Hospitalization for Low-Payment and High-Payment Hospitals

This supplementary material has been provided by the authors to give readers additional information about their work.

©2019 Krumholz HM et al. *JAMA Network Open*

eFigure 1. Construction of the Study Cohort: Heart Failure (Part A) and Pneumonia (Part B)

Part A

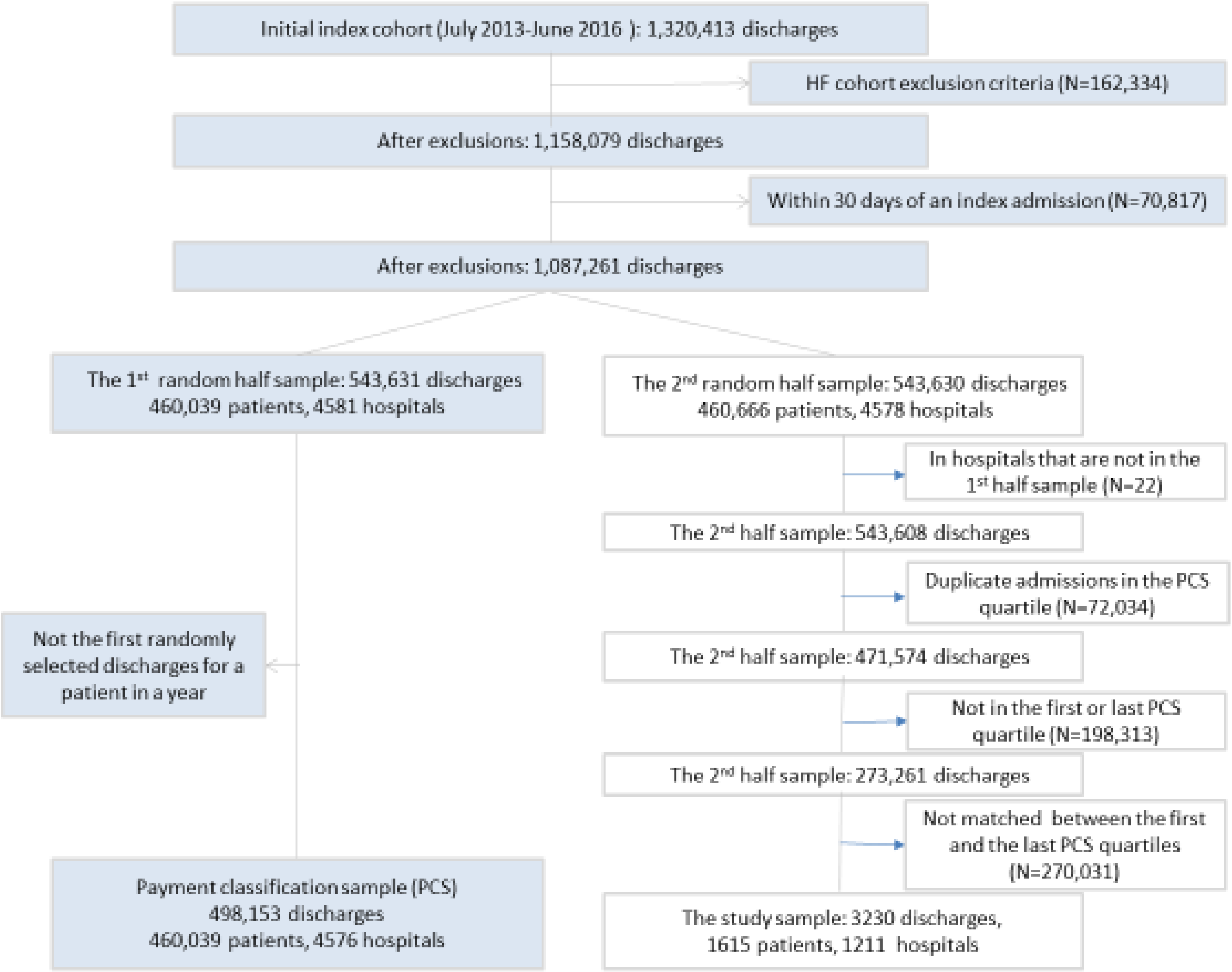

Part B

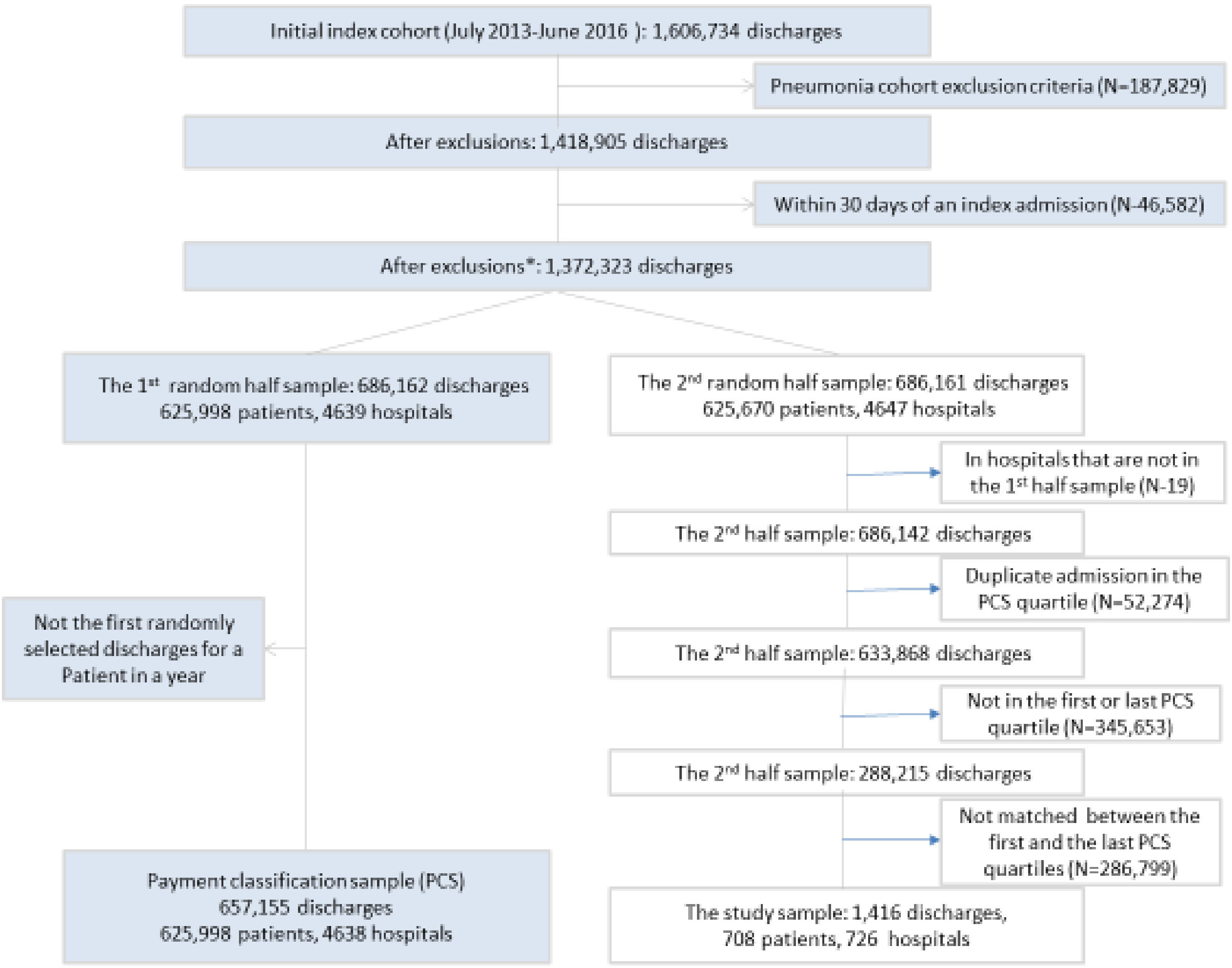

©2019 Krumholz HM et al. *JAMA Network Open*

eFigure 2. The Distribution of Hospital Risk-Standardized Payments in the Performance-Classification Sample

The solid curve is the normal density of the distribution.

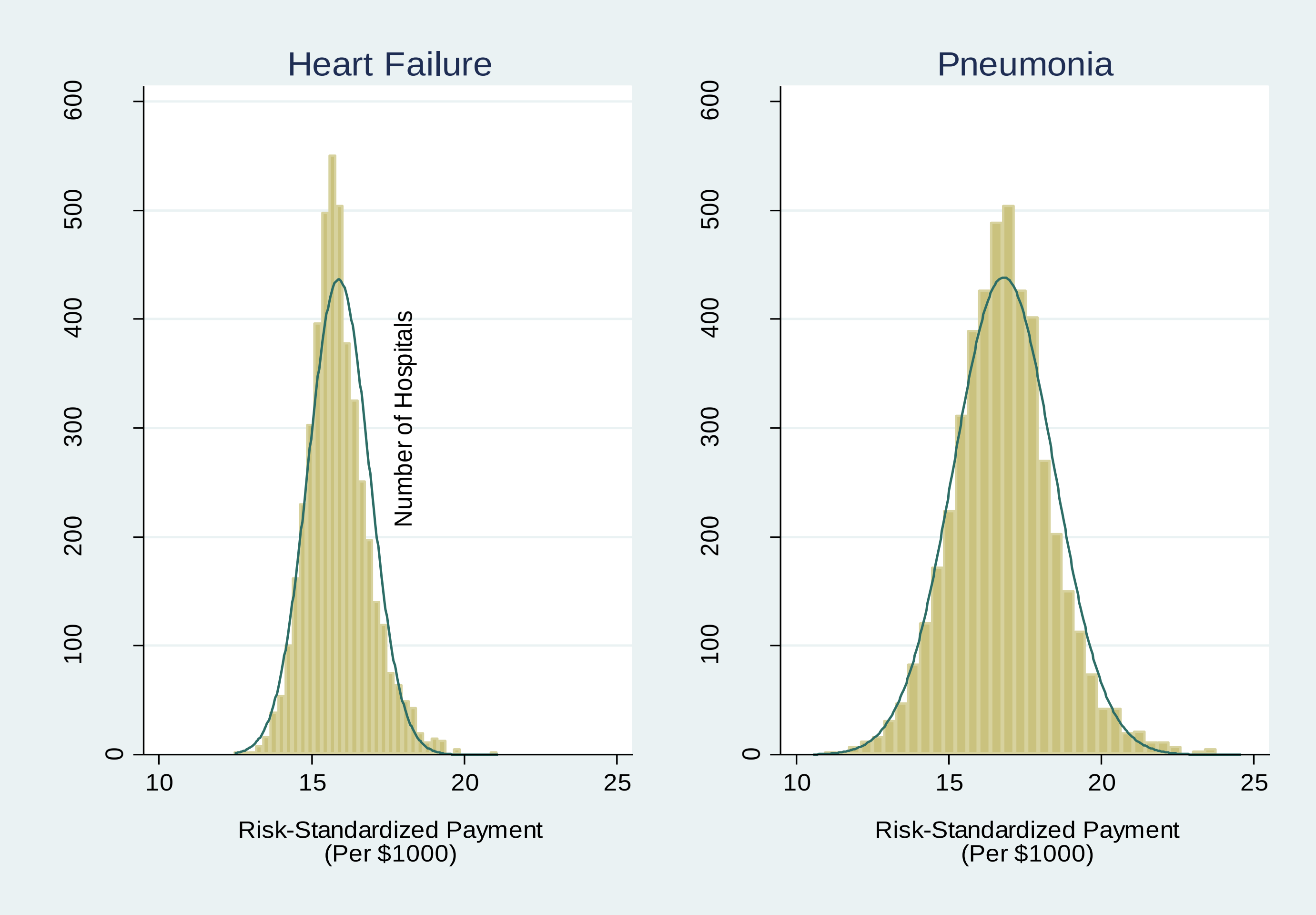

©2019 Krumholz HM et al. *JAMA Network Open*

eTable 1. Patient Admission Characteristics in the Heart Failure and Pneumonia Study Samples, Overall and in Low-Payment and High-Payment Hospitals

Description	Total		Admissions in Low-Payment Hospitals		Admissions in High-Payment Hospitals		Standardized Mean Difference
	n	%	n	%	n	%	
**Heart Failure**							
Number of admissions	3230	100.00	1615	100.00	1615	100.00	
Age (years)							
65-74	1138	35.23	576	35.67	562	34.80	0.0181
75-84	1270	39.32	634	39.26	636	39.38	-0.0025
≥85	822	25.45	405	25.08	417	25.82	-0.0171
History of infection	72	2.23	43	2.66	29	1.80	0.0587
Other infectious diseases	1363	42.20	666	41.24	697	43.16	-0.0389
Protein-calorie malnutrition	387	11.98	194	12.01	193	11.95	0.0019
Other significant endocrine and metabolic disorders	479	14.83	229	14.18	250	15.48	-0.0366
Obesity/disorders of thyroid, cholesterol, lipids	3015	93.34	1497	92.69	1518	93.99	-0.0522
Other gastrointestinal disorders	2290	70.90	1130	69.97	1160	71.83	-0.0409
Bone/joint/muscle infections/necrosis	89	2.76	49	3.03	40	2.48	0.0340
Other musculoskeletal and connective tissue disorders	2588	80.12	1279	79.20	1309	81.05	-0.0465
Delirium and encephalopathy	425	13.16	206	12.76	219	13.56	-0.0238
Dementia and senility	703	21.76	338	20.93	365	22.60	-0.0405
Schizophrenia/major depressive/bipolar disorders	270	8.36	132	8.17	138	8.54	-0.0134
Other psychiatric disorders	798	24.71	393	24.33	405	25.08	-0.0172
Respiratory arrest/cardiorespiratory failure/respirator dependence	1442	44.64	711	44.02	731	45.26	-0.0249
Angina pectoris/old myocardial infarction	2722	84.27	1348	83.47	1374	85.08	-0.0442
Heart infection/inflammation, except rheumatic	156	4.83	84	5.20	72	4.46	0.0347
Major congenital cardiac/circulatory defect	6	0.19	3	0.19	3	0.19	0.0000
Hypertension	2930	90.71	1465	90.71	1465	90.71	0.0000
Arrhythmias	2547	78.85	1258	77.89	1289	79.81	-0.0470
Cerebrovascular disease	788	24.40	383	23.72	405	25.08	-0.0317
Vascular or circulatory disease	1995	61.76	978	60.56	1017	62.97	-0.0497
History of pneumonia	1807	55.94	894	55.36	913	56.53	-0.0237
Other ear, nose, throat, and mouth disorders	1191	36.87	593	36.72	598	37.03	-0.0064
Dialysis status	176	5.45	85	5.26	91	5.63	-0.0164
Renal failure	2416	74.80	1192	73.81	1224	75.79	-0.0456
Decubitus ulcer of skin	222	6.87	111	6.87	111	6.87	0.0000
Chronic ulcer of skin, except decubitus	421	13.03	213	13.19	208	12.88	0.0092
Cellulitis, local skin infection	669	20.71	343	21.24	326	20.19	0.0260

©2019 Krumholz HM et al. *JAMA Network Open*

	Total		Admissions in Low-Payment Hospitals		Admissions in High-Payment Hospitals		Standardized Mean Difference
**Description**	**n**	**%**	**n**	**%**	**n**	**%**	
Hip fracture/dislocation	83	2.57	39	2.41	44	2.72	-0.0196
Internal injuries	66	2.04	32	1.98	34	2.11	-0.0088
**Pneumonia**							
Number of admissions	1416	100.00	708	100.00	708	100.00	
Age							
65-74	527	37.22	262	37.01	265	37.43	-0.0088
75-84	549	38.77	275	38.84	274	38.70	0.0029
≥85	340	24.01	171	24.15	169	23.87	0.0066
History of infection	60	4.24	27	3.81	33	4.66	-0.0420
Septicemia, sepsis, systemic inflammatory response syndrome/shock	372	26.27	190	26.84	182	25.71	0.0257
Other infectious diseases	639	45.13	318	44.92	321	45.34	-0.0085
Metastatic cancer and acute leukemia	64	4.52	29	4.10	35	4.94	-0.0408
Lung, upper digestive tract, and other severe cancers	127	8.97	62	8.76	65	9.18	-0.0148
Lymphatic, head and neck, brain, and other major cancers	168	11.86	85	12.01	83	11.72	0.0087
Benign neoplasms of skin, breast, and eye	165	11.65	75	10.59	90	12.71	-0.0660
Diabetes and DM complications	616	43.50	309	43.64	307	43.36	0.0057
Protein-calorie malnutrition	297	20.97	138	19.49	159	22.46	-0.0729
Other significant endocrine and metabolic disorders	146	10.31	74	10.45	72	10.17	0.0093
Liver disease	44	3.11	21	2.97	23	3.25	-0.0163
Gallbladder and biliary tract disorders	56	3.95	29	4.10	27	3.81	0.0145
Appendicitis	5	0.35	3	0.42	2	0.28	0.0238
Bone/joint/muscle infections/necrosis	37	2.61	21	2.97	16	2.26	0.0443
Osteoporosis and other bone/cartilage disorders	334	23.59	166	23.45	168	23.73	-0.0066
Severe hematological disorders	34	2.40	16	2.26	18	2.54	-0.0184
Disorders of immunity	109	7.70	53	7.49	56	7.91	-0.0159
Iron deficiency and other/unspecified anemias and blood disease	959	67.73	458	64.69	501	70.76	-0.1301
Delirium and encephalopathy	253	17.87	123	17.37	130	18.36	-0.0258
Dementia and senility	528	37.29	247	34.89	281	39.69	-0.0994
Drug/alcohol dependence/psychosis	89	6.29	43	6.07	46	6.50	-0.0174
Major psychiatric disorders	291	20.55	137	19.35	154	21.75	-0.0594
Hemiplegia, paraplegia, paralysis, functional disability	167	11.79	81	11.44	86	12.15	-0.0219
Neuropathy; muscular dystrophy	14	0.99	7	0.99	7	0.99	0.0000
Multiple sclerosis and Parkinson’s	109	7.70	55	7.77	54	7.63	0.0053

©2019 Krumholz HM et al. *JAMA Network Open*

	Total		Admissions in Low-Payment Hospitals		Admissions in High-Payment Hospitals		Standardized Mean Difference
**Description**	**n**	**%**	**n**	**%**	**n**	**%**	
Seizure disorders and convulsions	149	10.52	70	9.89	79	11.16	-0.0414
Coma, brain compression/anoxic damage	25	1.77	12	1.69	13	1.84	-0.0107
Polyneuropathy, mononeuropathy, and other neurological conditions/injuries	356	25.14	172	24.29	184	25.99	-0.0390
Respiratory arrest/cardiorespiratory failure/respirator dependence	654	46.19	326	46.05	328	46.33	-0.0057
Congestive heart failure	760	53.67	386	54.52	374	52.82	0.0340
Coronary atherosclerosis or angina	818	57.77	405	57.20	413	58.33	-0.0229
Heart infection/inflammation, except rheumatic	52	3.67	21	2.97	31	4.38	-0.0751
Valvular and rheumatic heart disease	407	28.74	198	27.97	209	29.52	-0.0343
Hypertensive heart disease	57	4.03	28	3.95	29	4.10	-0.0072
Stroke	166	11.72	77	10.88	89	12.57	-0.0527
Late effects of cerebrovascular disease, except paralysis	149	10.52	70	9.89	79	11.16	-0.0414
Chronic obstructive pulmonary disease	989	69.84	497	70.20	492	69.49	0.0154
Asthma	201	14.19	97	13.70	104	14.69	-0.0283
Pneumococcal pneumonia, empyema, lung abscess	127	8.97	62	8.76	65	9.18	-0.0148
Viral and unspecified pneumonia, pleurisy	1060	74.86	518	73.16	542	76.55	-0.0781
Pleural effusion/pneumothorax	393	27.75	189	26.69	204	28.81	-0.0473
Other respiratory disorders	914	64.55	457	64.55	457	64.55	0.0000
Other eye disorders	309	21.82	167	23.59	142	20.06	0.0855
Significant ear, nose, and throat disorders	38	2.68	19	2.68	19	2.68	0.0000
Other ear, nose, throat, and mouth disorders	607	42.87	296	41.81	311	43.93	-0.0428
Dialysis status	47	3.32	22	3.11	25	3.53	-0.0236
Urinary incontinence	170	12.01	91	12.85	79	11.16	0.0521
Other female genital disorders	55	3.88	27	3.81	28	3.95	-0.0073
Decubitus ulcer of skin	226	15.96	111	15.68	115	16.24	-0.0154
Vertebral fractures	75	5.30	37	5.23	38	5.37	-0.0063
Major fracture, except of skull, vertebrae, or hip	35	2.47	16	2.26	19	2.68	-0.0273
Internal injuries	15	1.06	6	0.85	9	1.27	-0.0414
Traumatic amputations and complications, and other injuries	686	48.45	324	45.76	362	51.13	-0.1075
Poisonings and allergic and inflammatory reactions	273	19.28	130	18.36	143	20.20	-0.0465
Major symptoms, abnormalities	1341	94.70	663	93.64	678	95.76	-0.0946
Minor symptoms, signs, findings	1346	95.06	675	95.34	671	94.77	0.0260

©2019 Krumholz HM et al. *JAMA Network Open*

eTable 2. Patient Demographics Between the Study Sample and the Exclusions in the Heart Failure and Pneumonia Cohorts

	Heart Failure					Pneumonia				
	Study Sample		Exclusions		*P*-value	Study Sample		Exclusions		*P*-value
	n	%	n	%		n	%	n	%	
Number of admissions	3230		540,400			1416		684,745		
Age					0.0000					*P*<0.0001
65-74	1138	35.23	143,140	26.49		527	37.22	196,662	28.72	
75-84	1270	39.32	198,741	36.78		549	38.77	249,846	36.49	
≥85	822	25.45	198,519	36.74		340	24.01	238,237	34.79	
Race					0.0000					0.3935
White	2510	77.71	449,643	83.21		1228	86.72	596,788	87.15	
Black	566	17.52	65,307	12.08		102	7.20	51,134	7.47	
Other	148	4.58	24,134	4.47		84	5.93	34,940	5.10	
Gender					0.0000					*P*<0.0001
Male	1638	50.71	246,785	45.67		802	56.64	320,147	46.75	
Female	1592	49.29	293,615	54.33		614	43.36	364,598	53.25	
Region					0.0000					*P*<0.0001
Northeast	520	16.10	114,208	21.13		192	13.56	127,017	18.55	
Midwest	924	28.61	134,165	24.83		352	24.86	170,047	24.83	
South	1284	39.75	220,539	40.81		704	49.72	279,715	40.85	
West	490	15.17	70,217	12.99		166	11.72	106,442	15.54	

©2019 Krumholz HM et al. *JAMA Network Open*

eTable 3. Diagnosis-Related Group Codes for the Index Hospitalization for Low-Payment and High-Payment Hospitals

Diagnosis-Related Group	Payment Weight	% of Admissions at Low-payment Hospitals	% of Admissions at High-Payment Hospitals
Heart Failure			
Circulatory disorders except AMI, w card cath w MCC (286)	2.1775	1.73	2.72
Circulatory disorders except AMI, w card cath w/o MCC (287)	1.1562	2.54	3.10
Heart failure & shock w MCC (291)	1.4809	35.17	39.01
Heart failure & shock w CC (292)	0.9707	40.80	35.60
Heart failure & shock w/o CC/MCC (293)	0.6737	14.43	10.03
Pneumonia			
Respiratory infections & inflammations w MCC (177)	1.9033	7.34	15.11
Respiratory infections & inflammations w CC (178)	1.3575	8.62	6.78
Simple pneumonia & pleurisy w MCC (193)	1.4261	15.82	19.07
Simple pneumonia & pleurisy w CC (194)	0.9695	34.89	20.62
Simple pneumonia & pleurisy w/o CC/MCC (195)	0.7111	13.98	7.06
Septicemia or severe sepsis w/o MV 96+ hours w MCC (871)	1.7926	14.55	23.45

©2019 Krumholz HM et al. *JAMA Network Open*
